# A Case of Successful Coil Embolization for a Late-Onset Type Ia Endoleak after Endovascular Aneurysm Repair with the Chimney Technique

**DOI:** 10.1155/2016/5307416

**Published:** 2016-09-06

**Authors:** Kimihiro Igari, Toshifumi Kudo, Takahiro Toyofuku, Yoshinori Inoue

**Affiliations:** Division of Vascular and Endovascular Surgery, Department of Surgery, Tokyo Medical and Dental University, Tokyo, Japan

## Abstract

Juxtarenal aortic aneurysms (JRAAs) are challenging to treat by endovascular aneurysm repair (EVAR) procedures. The chimney technique with EVAR (Ch-EVAR) is one of the feasible and less invasive treatments for JRAAs. However, the main concern of Ch-EVAR is the potential risk of “gutters,” which can lead to type Ia endoleak (EL). Most type Ia ELs after Ch-EVAR procedures occurred intraoperatively, and these ELs could be treated using an endovascular technique. However, late-onset type Ia ELs could be extremely rare, which might have a fear of conservative treatment. Type Ia ELs are associated with an increased risk of aneurysm rupture; therefore reintervention is recommended as soon as possible, and we should be aware of the occurrence of type Ia ELs after the Ch-EVAR procedure.

## 1. Introduction

Endovascular aneurysm repair (EVAR) is a widely accepted procedure in the treatment of infrarenal abdominal aortic aneurysms. However, the conventional EVAR technique is not suitable for treating juxtarenal aortic aneurysms (JRAAs) because it requires a minimum of 10–15 mm of healthy aorta in order to achieve an adequate sealing zone at the proximal neck. To make sure of enough proximal landing zone, some techniques for EVAR procedures have been developed.

Fenestrated and branched endografts (FBEs) have shown promising results with regard to the preservation of visceral perfusion [1]. However, the use of such customized devices mandates strict anatomical requirements, a manufacturing delay, and significant costs, and these devices are not commercially available in Japan. On the other hand, the chimney technique with EVAR (Ch-EVAR) can facilitate the performance of EVAR in the treatment of JRAAs. Ch-EVAR was originally reported by Greenberg et al. [2] as an adjunctive procedure involving visceral artery stenting during intentional endograft coverage of the vessel origin; it can establish an additional proximal fixation zone in patients with JRAAs. Most importantly, the components used in Ch-EVAR are commercially available, even in Japan. Some articles have therefore reported the operational efficiency of Ch-EVAR [3].

The main problem of Ch-EVAR is the risk of proximal type Ia endoleak (EL) due to so-called gutters. Gutters are channels that may appear between the main aortic endograft and the chimney graft. Gutter leakage after Ch-EVAR is relatively common; however, most ELs are resolved intraoperatively, and late-onset ELs, including type Ia ELs, have been treated conservatively [4]. We herein report the use of coil embolization in the treatment of a late-onset type Ia EL after Ch-EVAR for JRAA, which helped avoid an aneurysmal rupture.

## 2. Case Presentation

**Figure 1:**
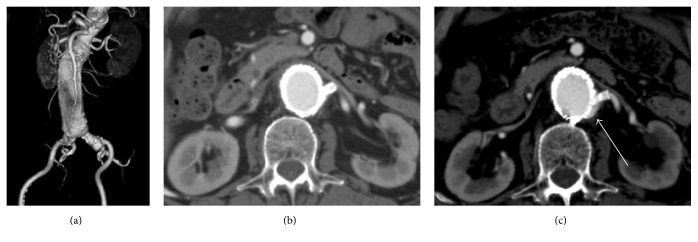
(a) Preoperative 3-dimensional computed tomography with the left anterior oblique view showed a juxtarenal aneurysm measuring 59 mm in diameter with a short proximal neck. (b) Early postoperative computed tomography showed a patent endograft and bare stent to the left renal artery without any endoleaks. (c) Two years after endovascular aneurysm repair, computed tomography showed the enlargement of the aneurysmal sac with a type Ia endoleak (white arrow).

A 77-year-old male with a history of cerebrovascular disease and chronic pulmonary obstructive disease (COPD) was referred to our institution to undergo treatment for a JRAA. A contrast-enhanced computed tomography (CT) scan revealed a JRAA of 59 mm in diameter with a short proximal neck (3 mm to the left renal artery) and a normal neck diameter (24.4 mm) (Figure 1(a)). Ch-EVAR was performed because the patient did not appear to be a good candidate for open aneurysmal repair due to his severe COPD. Main bifurcated endografts (Excluder™, W.L. Gore and Associates, Flagstaff, AZ, USA) with a 31 mm sized proximal diameter were positioned just below the ostium of the right renal artery, and a 6 mm bare metal stent (Express SD™, Boston Scientific, Cork, Ireland) was inserted and deployed in the left renal artery. Complete angiography showed a patent left renal artery and a patent endograft without ELs, including type Ia EL or any enhancement of the JRAA. A contrast-enhanced CT scan revealed good results in the early postoperative period (Figure 1(b)).

**Figure 2:**
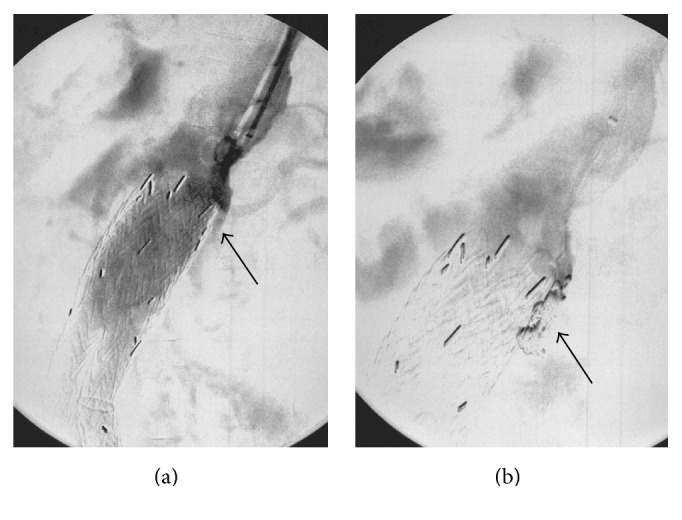
(a) Intraoperative angiography showed the origin of the type Ia endoleak (black arrow). (b) Intraoperative angiography after coil embolization showed the disappearance of the origin of type Ia endoleak (black arrow).

At the 1-year follow-up, a contrast-enhanced CT scan showed the shrinkage of the aneurysm (45 mm in diameter) with a patent left renal artery stent; however, the 2-year follow-up CT scan showed that the aneurysm diameter had grown (57 mm) and that a type Ia EL (Figure 1(c)) had occurred due to so-called gutters. He had no pulsation on his abdomen, and CT showed no change of aneurysmal neck (dilatation or shortening). A secondary procedure was therefore performed to treat the type Ia EL, which had caused the extension of the aneurysm. Under local anesthesia, a 4.5 Fr guiding sheath was inserted through the left brachial artery to cannulate the origin of the type Ia EL. Angiography showed a cavity, which caused and the route to the type Ia EL (Figure 2(a)). We thought that it would be impossible to reduce the type Ia EL using the “kissing balloon” technique; thus we attempted to perform coil embolization in the cavity. After gaining brachial access, a microcatheter was positioned into the cavity using a 0.014-inch guidewire. Thereafter, the cavity was embolized with two coils (Ruby™ Coil, Penumbra, Inc., Alameda, CA, USA). After coil embolization, the cavity was diminished. This contributed to the complete exclusion of the type Ia EL (Figure 2(b)). Three months after coil embolization, duplex ultrasonography showed no ELs, including the type Ia EL, and a patent left renal artery without aneurysmal enlargement.

## 3. Discussion

The current evidence concerning Ch-EVAR procedures shows that their clinical results are promising and that the incidence of perioperative morbidity and mortality, early mortality, and the occurrence of type Ia ELs does not differ to a statistically significant extent from that for FBE [5]. The chimney graft for Ch-EVAR could work well to confirm the endograft in the aortic wall. However, Ch-EVAR technique has a potential risk for the occurrence of type Ia ELs between the main endograft and chimney graft, so-called gutter leakage [6]. Furthermore, the occurrence of type Ia ELs is not always predictable. Coscas et al. [1] reported that 4 of 12 patients (30%) developed type Ia ELs during intraoperative Ch-EVAR procedures. Three of the 4 patients were treated using the kissing balloon technique; the other patient was treated using coil embolization; all type Ia ELs subsequently disappeared. During the follow-up period, 1 patient developed a new type Ia EL, which was carefully monitored. Most type Ia ELs that occurred intraoperatively were therefore treated simultaneously, while most late-onset type Ia ELs were not treated. Most type Ia ELs were observed to occur in the perioperative and early postoperative periods and often appeared to be sealed spontaneously. Even though the conservative management of type Ia ELs might be effective in some cases, we advocated that type Ia ELs should be treated as soon as possible, because they may result in aneurysmal rupture. In our case, the prompt treatment of the patient's type Ia EL led to a good outcome.

Several factors may contribute to the occurrence of type Ia ELs. Balloon-expandable or self-expandable stents with uncovered or covered stents might induce different reactions to lead to gutter leakage. A recent comparison between self-expandable and balloon-expandable stents as chimney stents demonstrated an increased tendency for type Ia ELs when self-expandable stents were used [7]. We therefore used balloon-expandable stents as chimney stents. Many authors have advocated that covered stents are beneficial because they reduce the pressurization of the gutters, lowering the risk of type Ia ELs [8]. However, other authors have suggested that bare stents are not inferior to covered stents with regard to renal patency and protection against type Ia ELs [9]. We have reported good results in the exclusion of AAAs with challenging neck anatomy by EVAR procedures [10] and JRAAs using bare chimney stents [11]. Furthermore, we can only use bare stents for Ch-EVAR because covered stents are not covered by Japanese National Health Insurance. Another factor associated with the occurrence of type Ia ELs is the new neck length. Donas et al. reported that patients with late-onset type Ia EL had a neck length <10 mm [12]. Most reports recommended that a new neck length of >20 mm was necessary in order to avoid type Ia ELs [6, 12]. Thus, we planned a new neck length of >10 mm with a bare stent, which led to the acceptable outcomes of our previous report [11].

The existing data have not provided firm conclusions as to whether these devices and techniques are associated with an increased risk of late-onset type Ia EL. However, type Ia ELs are associated with an increased risk of postprocedural aneurysm rupture; reintervention is therefore recommended as soon as possible after the diagnosis of a type Ia EL. Type Ia ELs after Ch-EVAR can be treated with concomitant ballooning of the stent grafts and visceral stents by the kissing balloon technique [1]. Even though this technique is feasible, it appears to be difficult and it is complicated to perform during the follow-up period. Thus, the simple coiling of the gutters has been described as a potential treatment for patients with gutter endoleaks [13]. In the present case, coil embolization completely resolved the late-onset type Ia EL. However, we should be aware that coils placed at the proximal site of the neck level present a significant hindrance to the interpretation of follow-up CT scans.

In conclusion, we herein described a case of late-onset type Ia EL after a Ch-EVAR operation, which was successfully treated by coil embolization. It is important to remain aware of the higher incidence of type Ia ELs after Ch-EVAR.
